# Enhancing counterfactual detection in multilingual contexts using a few shot clue phrase approach

**DOI:** 10.1038/s41598-025-96085-5

**Published:** 2025-04-10

**Authors:** Lekshmi Kalinathan, Karthik Raja Anandan, Jagadish Ravichandran, K. Devi, S. Benila, Abithkumar Ravikumar

**Affiliations:** 1 School of Computing Science and Engineering, VIT University, Chennai Campus, Rajan Nagar, Kelambakkam-Vandalur Road, Chennai, Tamil Nadu 600127 India; 2 Computer Science and Engineering, Sri Sivasubramania Nadar College Of Engineering, Rajiv Gandhi Salai, Kalavakkkam, Chennai, Tamil Nadu 603110 India

**Keywords:** Multilingual few-shot learning, Counterfactual detection, Clue-phrase integration, Cross-domain application, SetFit, Engineering, Electrical and electronic engineering

## Abstract

This research paper introduces an innovative counterfactual detection system, designed to tackle the complexities of identifying hypothetical statements that describe non-occurring events in diverse fields such as NLP, psychology, medicine, politics, and economics. Counterfactual statements, often encountered in product reviews, pose significant challenges in multilingual contexts due to the linguistic variations, and counterfactual statements are also less frequent in natural language texts. Our proposed system transcends these challenges by using a domain-independent, multilingual few-shot learning model, which significantly improves detection accuracy. Using clues as key innovation, the model demonstrates a 5–10% performance improvement over traditional few-shot techniques. Few-shot learning is a machine learning approach in which a model is trained to make accurate predictions with only a small amount of labeled data, which is particularly beneficial in counterfactual detection where annotated examples are scarce.The system’s efficacy is further validated through extensive testing on multilingual and multidomain datasets, including SemEval2020-Task5, with results underscoring its superior adaptability and robustness in various linguistic scenarios. The incorporation of clue-phrases during training not only addresses the issue of limited data but also significantly boosts the model’s capability in accurately identifying counterfactual statements, thereby offering a more effective solution in this challenging area of natural language processing.

## Introduction

Counterfactual statements are expressions that speculate about hypothetical scenarios or outcomes that did not actually occur. These statements often involve imagining alternative realities or events contrary to what actually happened. In the context of consumer reviews, counterfactual statements might involve hypothetical situations or outcomes that differ from the actual experiences described in the review. For example, a counterfactual statement in a review might speculate about how the product could have performed better if certain features were different or if a different decision had been made. Natural languages are replete with thought-provoking devices that can be challenging to comprehend and pose difficulties for language models. Counterfactual statements are one of those thought-provoking literary devices that is used to describe events that did not occur in the past or events that are hypothetical. These statements play a significant role in various fields and disciplines, including economics, politics, psychology, and sociology, among others.

Counterfactual statements are widely used in fields such as economics, politics, psychology, and medicine to study hypothetical scenarios and their impact. Economists use them to analyze policies or interventions in the economy^1^, while political analysts use them to study election results or political events^2^. In psychology, they are associated with various mental health problems^3^, while in medicine, they are used in medical decision-making and research on health outcomes. Counterfactual statements are used to compare different treatment options considering what would have happened if patients had received a different treatment^4^.

The prevalence of counterfactual statements in customer reviews poses a significant challenge, potentially misleading consumers about product features or choices that do not exist. Such misleading information can lead to a decrease in customer trust and base, particularly among those who lack language proficiency or specialized knowledge. Unfortunately, the limited ability of existing detection models for counterfactual statements to operate across multiple languages and domains exacerbates the problem. To solve this problem, a strong counterfactual detection system is suggested. It would use a few-shot learning model that works across domains and languages and was trained on well-known datasets like AMCFD. During training, language-specific clue phrases are added to help with data shortages and improve model performance. This offers a promising way to find and fix false information in customer reviews across a wide range of languages and topics.

## Related work

Recognizing the importance of counterfactual statements, Son et al.^5^ published a data set based on Twitter and Facebook posts. SemEval introduced the task of counterfactual statement detection in 2020^6^, after which Amazon released their dataset AMCFD based on product reviews^7^.

The following is a summary of the papers submitted for the SemEval 2020 conference task 5:

Ding et al., Fajcik et al. and Patil and Baths^8–10^ utilized transformer models like BERT for both subtasks. These transformers send the output tensor to a linear layer. The classification is then performed using either a sigmoid or a logistic regression model. This approach yields an F1 score of 80% and 70% for the respective subtasks. Using multi-stacked LSTM, Sung et al.^11^ achieved a performance of 80% in both subtasks. Similarly, Abi-Akl et al.^12^ adopted a cascaded BERT architecture and BiLSTM, achieving a performance of 79% in both subtasks. By adding more data to the BERT models and using its full power, Liu and Yu^13^ increased their success rates to 80.2% for both subtasks. A BERT model^14^, with 5-fold stratified cross-validation was used to give it an F1 score of 90%. The higher abstractions in the BERT model were also fine-tuned. The authors of^15^ built a simple neurosymbolic cloud network and trained it without the help of a person using RoBERTa and XLM. In two subtasks, they got 87% and 67% accuracy, respectively. A study^16^ used TF-IDF to improve different layers of BERT and found that the ensemble as a whole worked 85% of the time in the classification task. Using pre-trained BERT and token aggregation techniques, Lu et al.^16^ improved their performance to a 90% F1 score in both subtasks. Training ALBERTA in conjunction with the maximum ensemble, Li et al.^17^ achieved a score of 85% on the classification subtask. To perform the classification task, Tchiaze and Nouvel^18^ used SVM and an NLU-based model and obtained a success rate 80%, mostly due to the Rasa framework tool Rasa. Using conditional random fields with masked labels in hand, Bbvey et al.^19^ achieved a performance of 87%. Ou et al.^20^ got a performance score of 70% using a hierarchical attention network with ordered neurons, which is a method similar to that used in^21^. Most of the high-performing methods submitted to SemEval-2020 Task 5 used state-of-the-art pre-trained word embedding language models to represent sentences^9,16^.

The Spontaneous Counterfactual Inference (SCI) task, as introduced by^22^, offers a unique approach to studying counterfactual thinking, providing insight into memory processing and misattribution. By showing that people often remember making up counterfactuals as real events, it shows how memory can be affected by them, which has effects on decision-making and memory research. Researchers have investigated several different ways to find counterfactual statements. For example, Sung et al.^11^ used multistacked LSTM networks and got an impressive F1 score of 80%. Abi-Akl et al.^12^ were the first to combine BERT and BiLSTM in a cascade architecture, which received an impressive F1 score of 79%. Liu and Yu^13^ study demonstrated the efficacy of data enhancement in the refinement of BERT models, resulting in an improved F1 score of 80.2%. These advances highlight the evolving landscape of counterfactual detection methodologies to improve precision and efficacy in this domain.

In^23^, the authors used Bert-base and Roberta-base uncased for counterfactual classification and obtained F1 scores of 86.9% on the SemEval-2020 Task-1 dataset. Ojha et al.^24^ created a model for the extraction of linguistic characteristics with Roberta. They were able to obtain an F1 score of 91.18%. They also used traditional machine learning methods like Support Vector Machines (SVM) and Random Forests (RF), but with less success. Ding et al.^8^ uses Roberta with conditional random fields for counterfactual classification. The model achieved an F1 score of approximately 88%. 5-fold cross-validation was used to validate the performance achieved.

These studies show ongoing efforts to improve the accuracy of counterfactual statement detection. Researchers are trying different methods, from LSTM networks to combining BERT with BiLSTM and using data augmentation techniques, to improve natural language processing models in this area. These contributions highlight the evolving landscape of natural language processing, especially in tasks like detecting counterfactual statements. The advancements seen in SemEval-2020 Task-1 demonstrate the increasing effectiveness of machine learning models in tackling intricate linguistic tasks.

Some other notable works on counterfactual statements include the creation of the Amazon Multilingual Counterfactual Product Review Dataset (AMCFD) by O’Neil et al.^7^. O’Neil et al. created this data set in English, German, and Japanese, using clue phrases to identify commonly used words in counterfactual reviews. They conducted supervised transfer learning to evaluate performance. Fine-tuning the best performing CFD model in XLM-R increases the macro F1 score by 25% for German and 20% for Japanese target languages when compared to a model trained solely on data from the English source language. Similarly, Ushio and Bollegala^25^ used transfer learning with translation to target languages to achieve cross-lingual counterfactual classification, obtaining F1 scores of 73% and 82.9% in German and Japanese, respectively.

The prevailing focus in the aforementioned studies has predominantly been on word embeddings, a foundational aspect of many natural language processing models. However, there is an emerging paradigm shift toward the utilization of sentence embeddings, as highlighted in the seminal work by^26–28^. This approach represents a significant advancement in the field, as it seeks to capture the semantic essence of entire sentences rather than just individual words.

Reimers and Gurevych^28^ proposed a novel model to measure semantic similarity in text using sentence embeddings made by leading models such as BERT and RoBERTa. The method incorporates various pooling methods and uses a Siamese network architecture for training. The method allows us to fully understand text semantics by looking at cosine similarity between embeddings. This method, which has been applied to many languages and is now available to the public as “sbert,” is a big step forward in natural language processing. It makes it easier to process language in a way that takes context into account across a wide range of linguistic environments and shows a larger trend toward understanding larger textual contexts.

The study by^29^ introduces a notable advancement in few-shot learning, employing a Siamese network architecture alongside basic classifiers, such as logistic regression without prompts. Their method uses a Siamese network to fine-tune the model and machine learning classifiers for classification tasks, showing that it works well and efficiently with little training data. This approach showcases promising potential for few-shot learning applications, offering a streamlined alternative in the absence of extensive training data.

Guedes and da Silva^30^ describes a new way to use contrastive learning to make sentence-level embeddings of scientific articles. The technique should make it easier for NLP tasks to find semantic similarity. The method works better than traditional methods in classification and clustering tasks, as shown by experimental validation. However, more comparisons with cutting-edge methods would help the results even more. The study also discusses some important methods that have shaped the field, such as the relation network^31^, the prototypical network^32^, and the matching network^33^. These methodologies have been instrumental in advancing few-shot learning and were part of the experimental framework in Tunstall et al.’s research. Many new models have been added to the field of few-shot learning in natural language processing (NLP), including those by^34–36^. These models represent the forefront of research in NLP, employing innovative techniques to address the challenges of learning from limited data.

We have proposed a novel counterfactual detection system to effectively address challenges, distinguished by its domain independence and multilingual capabilities, crucial for broad applicability. Trained on datasets like the Amazon Counterfactual Product Review Dataset (AMCFD), it integrates language-specific clue phrases during training to overcome data limitations and enhance performance. This approach, which uses sentence embeddings in a few-shot learning framework, marks a significant advancement in counterfactual classification models. It promises increased accuracy and enables the precise identification of counterfactual statements, which is essential to extract reliable information from product reviews and textual data across languages.

## Methodology

### Prototypical Network

A prototype-based few-shot learning model^32^ using a Siamese network with mean distance as a classification metric is a type of machine learning that is used to handle tasks that need to be classified when there are not many examples for each class. The model combines two key components: a Siamese network and a prototype-based classification approach. The Siamese network is a neural network architecture that consists of two identical subnetworks that share the same weights. The subnetworks generate a feature representation for each of the two input samples, which they then compare to ascertain their similarity.The prototype-based approach to classification relies on the idea that the class of a new sample can be determined by finding the class prototype, which is a representative example of that class, that is closest to the sample in the feature space. The model computes the class prototypes by averaging the feature representations of all the examples in each class.At test time, the Siamese network computes the feature representation of a query sample. The mean of the feature representations is then computed for all the support samples belonging to each class, and the distance between the query sample’s feature representation and each class prototype. The class with the closest prototype is then predicted, as is the class of the query sample. Table 1 shows an experiment result with a three-way, three-shot scenario, which is the general configuration used in many papers. Surprisingly, even with the existence of a dense final layer, it seems to yield decent results that are comparable to the SetFit output in some cases.

**Table 1:** F1 scores from prototypical network and SetFit.

Three way-three shot						
Train-data	Prototypical network			SetFit		
	en-f1	de-f1	jp-f1	en-f1	de-f1	jp-f1
EN	0.28	0.39	0.18	0.27	0.53	0.19
DE	0.2	0.57	0.22	0.28	0.57	0.22
JP	0.12	0.37	0.13	0.23	0.79	0.19
EN-DE	0.09	0.4	0.14	0.29	0.61	0.15
EN-JP	0.2	0.6	0.15	0.24	0.69	0.20
DE-JP	0.24	0.4	0.4	0.27	0.70	0.19
all	0.24	0.6	0.4	0.26	0.69	0.21

### SetFit

SetFit^29^ is a few-shot framework that can be used for counterfactual detection by fine-tuning the Sentence Transformer model on a small number of labeled examples followed by training a classifier head on the embeddings generated from the fine-tuned Sentence Transformer. At inference time, the unseen example is passed through the fine-tuned Sentence Transformer, generating an embedding that when fed to the classification head outputs a class label prediction.

Note that, while SetFit can be used for counterfactual detection, the task of identifying counterfactuals is a complex and challenging problem, and the results may not always be accurate. Thus, in our modified SetFit (M-SetFit) architecture, we have introduced a novel strategy to include clue phrases during training to improve the performance of the counterfactual detection task.

### Few shot learning technique

The few-shot learning methodology proposed for counterfactual detection represents a novel and effective approach, as demonstrated in this study. Our method provides a domain-independent multilingual solution for finding few-shot counterfactuals, which allows the model to connect clue phrases in counterfactual statements correctly. This research focuses on how useful it is to pick out informative parts of sentences, called “clue-phrases,” and use them for classifier models in tasks that involve finding counterfactuals. Using clue phrases, our model achieves improved performance in identifying counterfactual statements, even in scenarios with limited training data. Furthermore, our findings suggest that this approach can be extended to enhance few-shot learning in general, particularly in domains where common clue phrases play a crucial role. By identifying and incorporating such clue phrases, our methodology can be adapted to various domains, offering a versatile and effective solution for a few-shot learning tasks.

### Proposed System

**Fig. 1:**
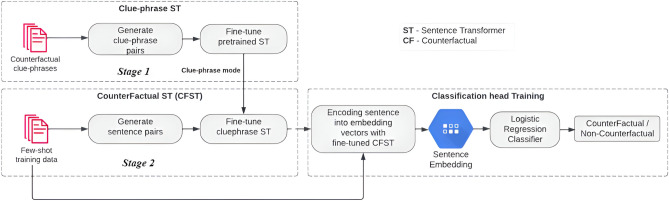
Proposed: Modified-SetFit.

The proposed system, M-SetFit, shown in Fig. 1, is an enhanced version of the SetFit model that includes the training of clues in addition to the existing model. The M-SetFit system consists of two main stages:

**Stage 1:** **Training on Clue-Phrases**

- The Sentence Transformer model is trained on clue phrases to compensate for the limited availability of counterfactual sentences. This stage focuses on recognizing similarities across languages by correlating clue phrases and similar words in their embeddings.

**Stage 2:** **Training on Counterfactual Statements**

- The weights from Stage 1 are used to train the counterfactual model. It is then trained using pairs of multilingual statements from the AMCFD reviews that are neither counterfactual nor not. The model learns the representations of these reviews in a joint embedding space, fine-tuning its weights to capture the differences between counterfactual and noncounterfactual reviews while preserving the clue-phrase knowledge from Stage 1.

The counterfactual model from stage 2 generates sentence embeddings in the final step, which we use to train the classifier head. A linear logistic regression model is chosen for classification due to its superior performance compared to other traditional machine learning classifiers, such as random forests and linear SVMs. This approach ensures that the proposed M-SetFit system accurately classifies data, enhancing the identification of counterfactual statements in multiple languages and domains.

The integration of clue-phrase training in stage 1 signifies a notable refinement within our model, which yields substantial improvements in its performance. Consequently, the proposed M-SetFit system introduces an advanced approach to constructing counterfactual detection models, harnessing clue-phrase models to enhance the precision of identifying counterfactual statements across various languages and domains. We attribute this improvement to the increased correlation between the clue phrases, which is discussed in further detail in the Results section.

### Architecture and training process

#### Model architecture

Our model consists of two main components:

- *Sentence transformer* Responsible for generating embeddings from the input text, fine-tuned on a small labeled dataset to adapt to counterfactual detection.
- *Classifier head* A simple feedforward neural network that takes the embeddings produced by the Sentence Transformer and outputs the final class prediction.

### Training process

1. *Fine-tuning* The Sentence Transformer is fine-tuned on the labeled dataset, integrating clue phrases into the training process by modifying the training data to highlight and emphasize the clue phrases indicative of counterfactuals.
2. *Embedding generation* After fine-tuning, the Sentence Transformer generates embeddings for each example in the dataset, capturing the semantic information of the sentences with an emphasis on clue phrases.
3. *Classifier training* The embeddings are then used to train the classifier head, which learns to distinguish between counterfactual and non-counterfactual statements based on the embeddings.
4. *Evaluation and testing* The trained model is evaluated on a separate test set using metrics such as accuracy, precision, recall, and F1 score to assess its performance.

Consequently, the proposed M-SetFit system provides an improved approach to create models to detect counterfactual statements using clue-phrase models. This makes it easier to identify counterfactual statements in multiple languages and domains.

## Experiments

### Setup

#### Dataset

The dataset created by Yang et al. was used for the SemEval-2020 Task-5 Counterfactual Recognition, which contains counterfactual data from various domains such as politics, economics, and healthcare. It includes **738 counterfactual statements** out of **7000 statements**, making counterfactual instances significantly lower than non-counterfactual ones. An example of a counterfactual statement from this dataset is:

“If the government had acted sooner, the crisis could have been avoided.”

This dataset is used to demonstrate how our proposed system performs across multiple domains.

Additionally, we used the **Amazon Multilingual Counterfactual Product Reviews Dataset (AMCD)**, which was collected by^7^. This dataset contains customer reviews in **English, German, and Japanese**, with counterfactual reviews being significantly lower than non-counterfactual ones. To illustrate the nature of the counterfactual data, here are two sample statements from the dataset:

“I would have bought this shirt if it were available in red.”

“I wish I would have loved this one, but I didnâ€™t.”

The distribution of the dataset is shown in Table 2.

**Table 2:** Dataset used.

Dataset	Language	Training		Testing	
		Total	Counterfactual	Total	Counterfactual
Amazon CFD dataset	EN	7176	824	1195	139
	DE	5600	3865	934	650
	JP	5600	525	934	96
Sem Eval	EN	-	-	7000	738

The distribution of the dataset also affected the selection of hyperparameters. In our suggested M-SetFit system, we used a two-stage training strategy because the number of counterfactual assertions is much smaller. In the second stage, we improve the model in the counterfactual dataset to enhance its classification performance. This phase comes after the first stage trains the model on clue phrases to collect useful contextual information. This approach guarantees the model’s strong cross-language and cross-domain generalization.

### Training strategy and model

We refined the **paraphrase-multilingual-mpnet-base-v2** model for our experiments, which is a sentence transformer that is specifically designed to detect multilingual paraphrases. This model was selected for its robust multilingual performance and strong semantic representation capabilities.

A two-stage training strategy was implemented. In the initial phase, we trained the model on a dataset that contained clue phrases to extract contextual information. The model was fine-tuned in the second stage to enhance classification accuracy by focusing on counterfactual samples.

We used a ** few-shot learning approach**, training with a batch size of 3 for 10 epochs using cosine similarity loss. The model was then used to produce sentence embeddings, which were subsequently classified using a ** logistic regression classifier**. This classifier was selected for its superior performance in comparison to conventional classifiers such as Random Forest and SVM.

The model was trained in a high performance computing configuration that included **2 Ã- NVIDIA H100 80GB PCIe 5.0 x16 GPUs**, ensuring that the training process was efficient and free of computational constraints.

### Hyperparameters

To ensure robust counterfactual detection, we experimented with multiple sentence transformers and selected **paraphrase-multilingual-mpnet-base-v2** due to its strong semantic representation capabilities and multilingual adaptability.

Several models were evaluated:

- *paraphrase-multilingual-mpnet-base-v2* Chosen for its superior ability to capture sentence semantics, especially in multilingual paraphrase detection tasks.
- *stsb-xlm-r-multilingual* Primarily trained for semantic similarity, but lacks domain-specific optimization for counterfactual classification.
- *paraphrase-multilingual-MiniLM-L12-v2* A lightweight alternative with lower computational overhead but slightly lower classification performance.

Given our high-performance computing setup (**2 Ã- NVIDIA H100 80GB PCIe 5.0 x16 GPUs**), the computational cost was not a limiting factor. Thus, we prioritized **classification accuracy and contextual representation** over efficiency.

We trained the clue-phrase model with a batch size of 10 over 2 epochs, while the sentence model training was conducted with a batch size configuration of 2. The necessity to effectively train the model on a short dataset while avoiding overfitting served as the basis for the hyperparameter selection. While the sentence model’s batch size of two guarantees fine-tuning stability on a small labeled dataset, the clue-phrase model’s batch size of ten permits stable updates during training. To avoid overfitting to the sparse counterfactual samples, the number of epochs was kept small.

Because it performed better than other conventional classifiers like Random Forest and Linear SVM, a Linear Logistic Regression classifier was chosen for the last classification step. This model efficiently uses the embeddings produced by the refined sentence transformer to ensure robust counterfactual detection.

By carefully integrating and optimizing hyperparameters, the M-SetFit system learns counterfactual representations efficiently and maintains strong generalization capabilities across diverse languages and domains. This makes it a powerful tool for various NLP tasks, enabling effective adaptation and performance in multilingual and multi-domain scenarios.

### Results

The model results shown below has a greater improvement over the standard few-shot learning algorithms like SetFit.

The following experiments are conducted to determine the efficiency of our proposed system. The experiments are conducted on clue-phrase detection, which extends its support to the superiority of our model. Subsequently, the model has been tested on multilingual scenarios as well as on the multi-domain SemEval2020-Task5 dataset.

#### Clue-phrase analysis

**Fig. 2:**
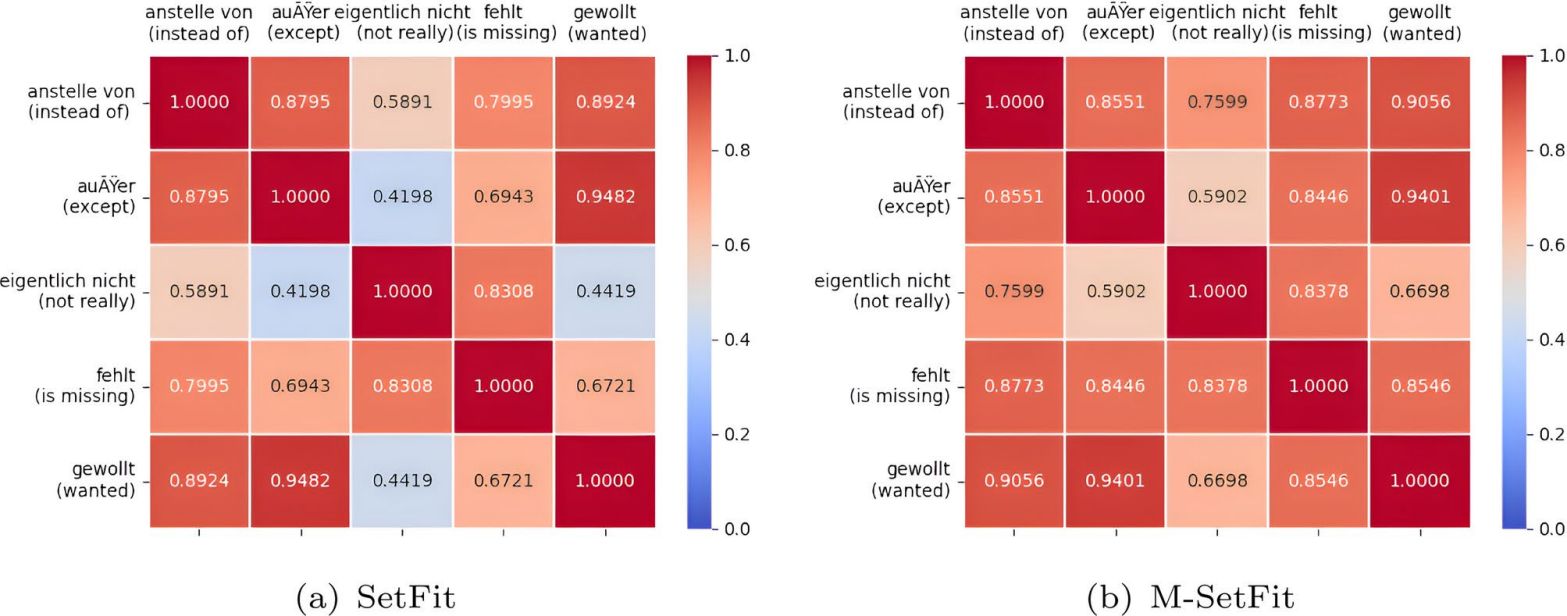
Correlational analysis of German.

**Fig. 3:**
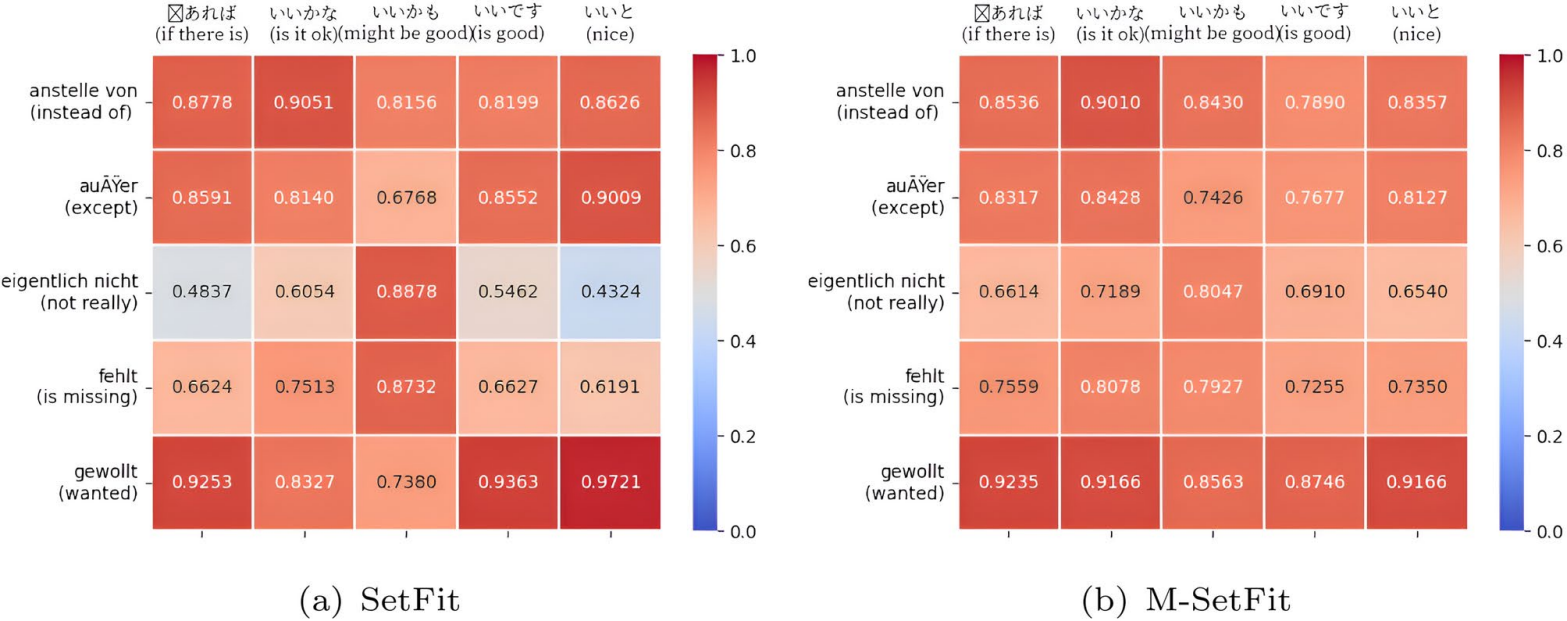
Correlational analysis of German and Japanese.

Heatmaps serve as a tool to illustrate the correlation between cluephrase embeddings. A comparison between the cluephrase embeddings generated by SetFit and M-SetFit visually is shown in the heatmap in Figs. 2 and 3. The correlation analysis of all possible combinations between English, German, and Japanese is performed. We list two sample heatmaps from the analysis to demonstrate the superior performance of our model.

The correlation heatmaps in Figs. 2 and 3. illustrate the similarity between clue phrases in German and across German-Japanese pairs. In Fig. 2, where both axes represent German clue phrases, the M-SetFit model demonstrates stronger correlations than SetFit, indicating its ability to capture closer relationships between German phrases. This improved representation makes it easier for the classifier to distinguish and categorize German sentences accurately. Similarly, in Fig. 3, where German clue phrases are compared with their Japanese counterparts, M-SetFit again exhibits higher similarity values, suggesting that it effectively aligns bilingual clue phrases. This enhanced cross-lingual representation facilitates better classification of both German and Japanese sentences, ultimately improving multilingual performance.

#### Evaluation efficacy of M-SetFit

The proposed model M-SetFit has been tested on Multi-Domain and Multilingual datasets.

##### Tested on AMCFD (multilingual)

We tested how well the counterfactual model worked in a multilingual setting using the AMCFD dataset. The results can be seen in Tables 3, 4 and 5.

It presents a comparison of one-shot, three-shot, and five-shot performance for different language pairs using two different training models, SetFit and M-SetFit, respectively. We display the F1 scores for seven distinct language pairs, namely English (EN), German (DE), and Japanese (JP), along with their respective combinations. Each table provides insights into how increasing the number of shots affects performance. The highest F1 scores for each language pair are highlighted in bold.

**Table 3:** One-shot performance comparison in multilingual setup.

Training	F1-scores of one-shot setup					
	SetFit			M-SetFit		
	EN	DE	JP	EN	DE	JP
EN	0.368	0.644	0.203	0.361	0.659	0.208
DE	0.312	0.573	0.179	0.344	**0.681**	0.198
JP	0.349	0.626	0.204	**0.390**	0.632	0.225
EN-DE	0.363	0.649	0.211	0.384	0.614	0.231
EN-JP	0.380	0.627	0.220	0.396	0.653	0.228
DE-JP	0.317	0.577	0.186	**0.388**	**0.656**	0.213
EN-DE-JP	0.375	0.598	0.217	**0.411**	**0.652**	0.242

**Table 4:** Three-shot performance comparison in multilingual setup.

Training	F1-scores of three-shot setup					
	SetFit			M-SetFit		
	EN	DE	JP	EN	DE	JP
EN	0.454	0.636	0.27	0.423	0.619	0.254
DE	0.412	0.693	0.243	0.419	0.69	0.23
JP	0.382	0.602	0.222	0.325	0.569	0.215
EN-DE	0.408	0.684	0.225	0.46	0.689	0.281
EN-JP	0.441	0.59	0.249	0.454	**0.652**	0.491
DE-JP	0.411	0.646	0.235	0.451	0.644	0.24
EN-DE-JP	0.487	0.75	0.313	**0.529**	0.74	0.32

**Table 5:** Five-shot performance comparison in multilingual setup.

Training	F1-scores of five-shot setup					
	SetFit			M-SetFit		
	EN	DE	JP	EN	DE	JP
EN	0.32	0.70	0.19	0.374	0.755	0.073
DE	0.67	0.60	0.68	0.379	0.815	0.204
JP	0.37	0.57	0.46	0.349	0.751	0.191
EN-DE	0.74	0.69	0.65	0.491	0.577	0.266
EN-JP	0.67	0.71	0.57	0.576	**0.784**	0.327
DE-JP	0.69	0.70	0.56	0.414	**0.805**	0.255
EN-DE-JP	0.87	0.76	0.66	**0.638**	0.68	0.33

Tables 3 and 4 illustrate that M-SetFit typically surpasses SetFit, especially when the training data comprise clue phrases, thus affirming its effectiveness in few-shot learning tasks. The implementation of the five-shot configuration in Table 5 further investigates the effect of increasing the number of training examples.

The five-shot results indicate that performance does not improve consistently across all language pairs. Some combinations demonstrate significant improvements in F1 scores (e.g. DE-JP and EN-JP under M-SetFit), while others display variability, with declines observed in some instances (e.g. JP in EN configuration). This indicates that although more training samples can improve model generalization, there may be diminishing returns or overfitting effects contingent upon the language pair and domain complexity.

A notable finding from Table 5 is the substantial improvement in the F1 score for the German language (DE) in several training configurations under M-SetFit, achieving 0.815 when trained on DE data. Likewise, EN-JP training results in an improvement of the F1 score for the DE language, underscoring that utilizing additional data points increases model adaptability. However, certain instances, such as the JP-only configuration, exhibit a decline in performance, perhaps attributable to domain shifts or data imbalance effects.

Finally, our findings suggest that increasing the number of shots beyond three-shot learning yields mixed effects: improving generalization in certain instances but causing inconsistencies in others. When scaling a few-shot learning technique, it is important to have a balanced and diverse data set because the effectiveness of five-shot training depends on the linguistic combination and the caliber of the available samples.

##### Tested on SemEval2020Task5 (multi-domain)

The results of the counterfactual model test on an unseen multi-domain dataset are displayed in Table 6. The evaluation of our modified model for the SemEval dataset is in contrast to that of a state-of-the-art few-shot model. The suggested model performs better than the current model, even in a multi-domain configuration, as is readily apparent.

**Table 6:** One-shot, three-shot, and five-shot performance comparison in multidomain setup.

	One-shot		Three-shot		Five-shot	
Training	SetFit	M-SetFit	SetFit	M-SetFit	SetFit	M-SetFit
EN	0.204	0.208	0.240	0.251	0.223	0.202
DE	0.188	0.199	0.213	0.226	0.291	0.222
JP	0.197	0.218	0.215	0.190	0.245	0.192
EN-DE	0.218	0.194	0.228	0.246	0.245	0.229
EN-JP	0.201	0.212	0.234	0.240	0.275	0.252
DE-JP	0.218	0.211	0.234	0.235	0.318	0.236
EN-DE-JP	0.206	0.222	0.241	0.260	0.302	0.327

Table 6 presents a comparison of F1 scores in a configuration of one shot, three shot, and five shots for a multidomain scenario that is comparable to the multilingual analysis conducted in the previous section. In this table are presented different training setups and their respective F1 ratings for the SetFit and M-SetFit methods. It offers a comprehensive understanding of the efficacy of various language combinations in a multidomain environment. In both multilingual and multidomain configurations, the M-SetFit model generally outperforms the SetFit model, as evidenced by its higher F1 scores across all languages and domains. This evidence indicates that the model’s performance in few-shot learning tasks can be enhanced by incorporating clue phrases into the training approach.

Furthermore, the hypothesis that the generalization of the model is improved by the incorporation of a five-shot setup is further supported by the additional improvement in F1 scores. This enhancement is due to the fact that the model is able to more effectively capture domain-specific variations and domain-invariant patterns as a result of the more diverse representation of counterfactual and non-counterfactual instances provided by the additional training samples. The model’s capacity to generalize to unseen data is refined, and overfitting is mitigated by the increased sample size, which is particularly effective in the multi-domain context, where variations across domains can introduce significant challenges.

The noted enhancement in performance with five-shot training substantiates the assertion that few-shot learning models gain from supplementary data points, until a specific threshold is attained, beyond which returns diminish. The results suggest that the use of a five-shot method is warranted, as it yields more consistent and reliable model predictions in the context of the unknown SemEval dataset. Further work, including testing with seven-shot or ten-shot configurations, may yield an enhanced understanding of the ideal equilibrium between data efficiency and model performance.

## Discussion

### Correlational analysis of German

As seen in Fig. 2., the horizontal and vertical axes are German clue phrases. The heatmap depicts the similarity between the corresponding pairs of clue phrases in German. According to the heatmaps, the M-SetFit model showed more similarity between German cluephrases than the SetFit model, indicating that the M-SetFit model can relate German cluephrases closer to each other.This evidence indicates that M-SetFit relates German clue phrases more closely, making it easier for the classifier head to classify German sentences.

### Correlational analysis between German and Japanese

As seen in Fig. 3., the horizontal axis consists of German cluephrases, and the vertical axis consists of Japanese cluephrases. The heatmap illustrates the degree of similarity between the corresponding pairs of German and Japanese cluephrases.

According to the heatmaps, the M-SetFit model showed more similarity between German and Japanese cluephrases than the SetFit model, indicating that the M-SetFit model can relate the bilingual cluephrases closer to each other.The result indicates that M-SetFit relates the German and Japanese cluephrases more closely, making it easier for the classifier head to classify both German and Japanese sentences.

### Correlation analysis and performance improvement

The proposed M-SetFit model outperforms SetFit in capturing semantic relationships among clue phrases in both monolingual and multilingual contexts, as evidenced by the correlation heatmaps in Figs. 2 and 3.

#### Findings from Fig. 2 (German Clue-Phrase Correlation)

The heatmap in Fig. 2 illustrates the pairwise similarity scores between German clue phrases. The following key observations highlight the superior performance of M-SetFit:

- The M-SetFit model demonstrates more significant correlation values between semantically related phrases, including *“außer (except)”* and *“gewollt (wanted)”*, than SetFit.
- It is inferred that M-SetFit is more adept at capturing linguistic subtleties, thereby establishing a more coherent semantic space for German clue phrases.
- By enhancing the aggregation of similar clue phrases, the classifier head is able to more effectively differentiate between counterfactual and non-counterfactual statements.

#### Findings from Fig. 3 (German-Japanese Cross-Lingual Correlation)

In multilingual scenarios, the correlation heatmap in Fig. 3 reveals that:

- The M-SetFit model demonstrates a higher degree of similarity between the corresponding German and Japanese clue phrases, including *“an Stelle von (instead of)”* and
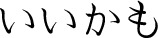
*(might be good)*
- SetFit demonstrates diminished and more dispersed correlation values, which indicates a weaker correspondence between the two languages.
- This indicates that M-SetFit offers superior cross-lingual mapping, enhancing generalization across multilingual datasets.

#### Implications of the correlation analysis

The stronger intra-language and cross-language correlations observed in M-SetFit contribute to:

- A more refined embedding space, leading to enhanced few-shot learning performance.
- Improved multilingual generalization, as the model better captures relationships across languages.
- Higher classification accuracy by ensuring that similar clue phrases remain closer in the embedding space, reducing ambiguity in counterfactual detection.

These results further validate the efficacy of M-SetFit for cross-lingual counterfactual categorization and support the benefit of our suggested method in both monolingual and multilingual contexts.

## Conclusion

The few-shot learning methodology proposed for counterfactual detection represents a novel and effective approach, as demonstrated in this study. Our method provides a domain-independent multilingual solution for finding few-shot counterfactuals, which allows the model to connect clue phrases in counterfactual statements correctly. This study focuses on how useful it is to pick out useful parts of sentences, called “clue phrases,” and use them as classifier models in tasks that involve finding counterfactuals. Using clue phrases, our model achieves improved performance in identifying counterfactual statements, even in scenarios with limited training data. Although the novel method shows significant potential, the paper would benefit from a clearer explanation of how it differs from and improves existing techniques in counterfactual detection. Future research should focus on elucidating these aspects to strengthen the argument for the superiority of the method and its broader applicability. Furthermore, our findings suggest that this approach can be extended to enhance few-shot learning in general, particularly in domains where common clue phrases play a crucial role. By identifying and incorporating such clue phrases, our methodology can be adapted to various domains, offering a versatile and effective solution for a few-shot learning tasks. In summary, this study underscores the importance of using clue phrases for effective counterfactual detection and highlights the potential of our approach to improve few-shot learning in different domains and languages.

## Data Availability

The dataset of Amazon multilingual counterfactual product reviews used in this investigation was collected from the publication referenced as^7^.
